# Corrosion Resistance of Graphene oxide/Silver Coatings on Ni–Ti alloy and Expression of IL-6 and IL-8 in Human Oral Fibroblasts

**DOI:** 10.1038/s41598-020-60070-x

**Published:** 2020-02-24

**Authors:** Viritpon Srimaneepong, Dinesh Rokaya, Pasutha Thunyakitpisal, Jiaqian Qin, Kanokwan Saengkiettiyut

**Affiliations:** 1 Department of Prosthodontics, Faculty of Dentistry, Chulalongkorn University, Bangkok, Thailand; 2 Research Unit of Herbal Medicine, Biomaterials and Materials for Dental Treatment, Faculty of Dentistry, Chulalongkorn University, Bangkok, Thailand; 3 Department of Anatomy, Faculty of Dentistry, Chulalongkorn University, Bangkok, Thailand; 4 Metallurgy and Materials Science Research Institute (MMRI), Chulalongkorn University, Bangkok, Thailand

**Keywords:** Dental biomaterials, Dental implants, Preclinical research

## Abstract

Graphene based materials (GBMs) have potentials for dental and medical applications. GBMs may cause changes in the levels of cytokine released in the body. This study aimed to study the corrosion resistance of graphene oxide (GO) and GO/silver (GO/Ag) nanocomposite coated nickel-titanium (NiTi) alloy by electrophoretic deposition and to access the viability of human pulp fibroblasts, and the interleukin (IL)-6 and IL-8 expression level. The bare and coated NiTi samples were characterized by scanning electron microscope (SEM), energy-dispersive X-ray spectroscopy (EDS), Raman spectroscopy, surface profilometry, and X-ray diffraction (XRD). The corrosion resistance of the bare NiTi and coated NiTi samples were investigated by potentiodynamic polarization and electrochemical impedance spectroscopy in 3.5% NaCl solution. The cell viability of human pulp fibroblasts was accessed by the treated culture medium of the bare NiTi and coated NiTi alloys containing 1% fetal bovine serum. IL-6 and IL-8 expression levels were studied by human enzyme-linked immunosorbent assay (ELISA). Data were analyzed using One-way ANOVA (α  =  0.05). Both the GO-coated NiTi and GO/Ag-coated NiTi alloys showed better corrosion resistance, a lower rate of corrosion, and higher protection efficiency than the bare NiTi alloy. The coated NiTi alloys were biocompatible to human pulp fibroblasts and showed upregulation of IL-6 and IL-8 levels.

## Introduction

Nickel-titanium (NiTi) alloy is widely used due to the special properties of superelasticity and shape memory. Common biomedical applications of NiTi alloys include vascular stents, staples, catheter guide wires, orthodontic wires, and endodontic instruments. However, NiTi alloy exhibits corrosion attack compared to stainless steel, cobalt-chrome, or β-titanium and it results in Ni and Ti ions release. It was found that a significant release of Ni and Ti ions from dental alloys in corrosive environment is noted although the amount of Ni ions release diminish with time^1^. Another study found that Ni leaching occurred after placement of the NiTi orthodontic archwires, bands and brackets and was associated with an increase of the Ni ion concentration in the patient’s saliva which lasted for 10 weeks and then decreased slowly^2^. These ions can cause foreign body reactions, allergy, and adverse reactions in the human body, such as stomatitis, burning sensation, angular cheilitis, and loss of taste^3,4^. Similarly, Ni along with Co and Cr remains the most common metals associated with surgical implant failure due to metal sensitization^5^. Therefore, the surface modifications and/or coatings have an important role in surface improvement and reducing the corrosion and body reactions. Although various polymer coatings have been tried on NiTi alloy, there has always difficult in making a successful coatings^6,7^. Graphene is a sheet of sp^2^ carbon atoms that creates a 2-D hexagonal honeycomb structure^8^. Graphene based materials (GBMs) have been used for fabricating various nanocomposite coatings to improve surface and mechanical properties, such as strength, durability, corrosion resistance, stability, and friction^8–12^. Importantly, anti-corrosion property of graphene-based nanocomposites is useful for biomedical applications, such as in wires, prostheses, and stents^12,13^. Graphene can be incorporated with various elements and polymers to produce nanocomposites for environmental, antibacterial, biosensors, and coating applications^9,11,14–16^. Electrophoretic deposition (EPD) is a popular technique to produce a thin uniform coatings of graphene composites with excellent mechanical and surface properties for various applications.

In terms of biocompatibility, cytokines are associated with the modulation of inflammatory processes and immune system. One of the important components of the immune system is interleukin (IL)-6 and IL-8. IL-6 is a cytokine of both innate and acquired immunity, and its main cellular action is to stimulate the growth of B lymphocytes to differentiate into antibody-producing cells^17^. IL-8 acts as a chemoattractant for neutrophils in the inflammatory sites^18^. Enzyme-linked immunosorbent assay (ELISA) is a specific, precise, and convenient method for measuring macromolecular protein and polysaccharide^19^. The aims of this study were to study the corrosion resistance of graphene oxide (GO) and GO/silver (GO/Ag) nanocomposite coated nickel-titanium (NiTi) alloy by electrophoretic deposition and evaluate, to access the viability of human pulp fibroblasts (HPFs), and the IL-6 and IL-8 expression level.

## Results and Discussion

**Figure 1:**
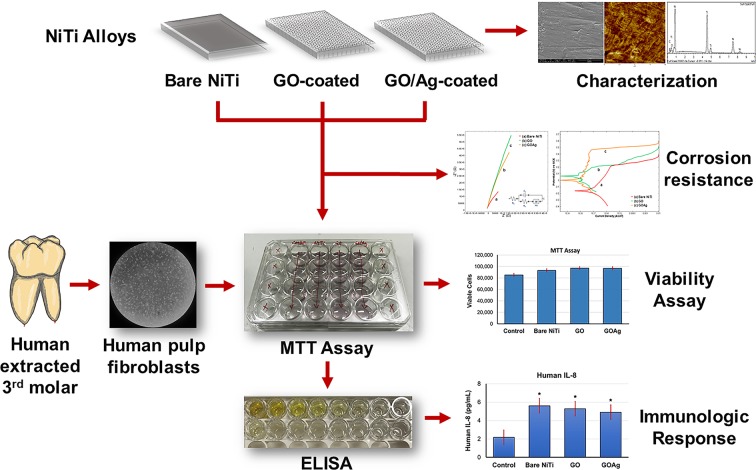
The overview of the study.

NiTi alloys are subjected to corrosive attack in the human body with the release of Ni and Ti ions due to physiological aqueous environments^1,20^. GBMs have been used studied as a potential coatings materials using various techniques on various metals, such as Cu, Zn, Ti, and NiTi and they have been found valuable for the protection against the corrosion^13,14,21–25^. In this study, we used GO and GO/Ag as a thin, biocompatible, and antimicrobial coating materials on NiTi alloy. Atomically smooth graphene and GO has been used as an inert and biocompatible surface coating material, and has potential to improve implant properties such as stent, prosthetic implant, and orthodontic wires^8,9,21,23^. The overview of the study is shown in Fig. 1. NiTi alloy substrates were coated with GO-coatings and GO/Ag-coatings. The coatings were characterized and tested for their corrosion resistance, biocompatibility, and immunologic response which are discussed as follows.

### Characterizations

**Figure 2:**
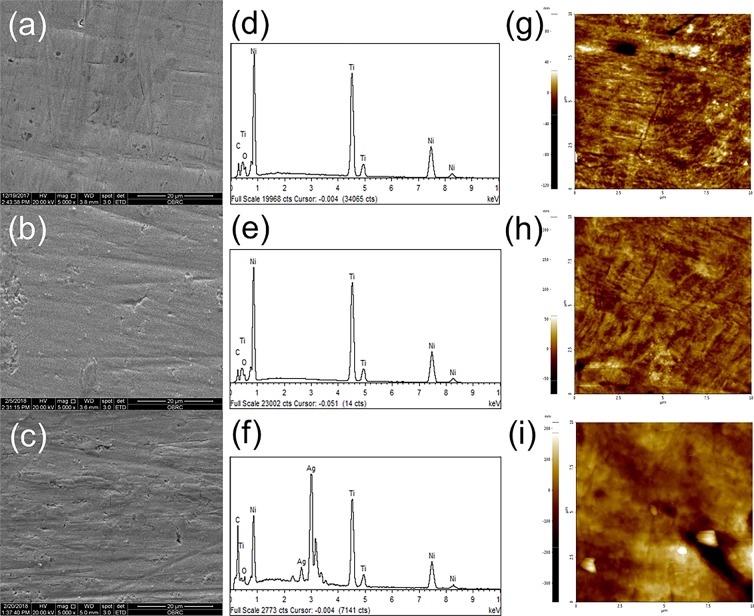
(**a**–**c**) SEM images, (**d**–**f**) EDS analysis, and (**g**–**i**) AFM images of the bare NiTi, GO-coated NiTi, and GO/Ag-coated NiTi alloy, respectively.

**Figure 3:**
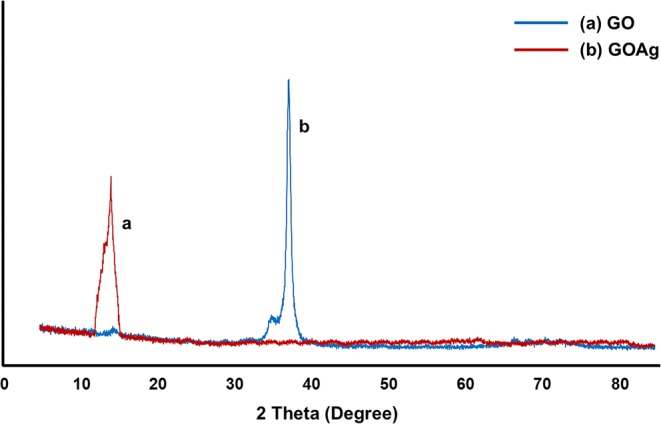
X-ray diffraction patterns of the GO-coated NiTi (GO) and GO/Ag-coated NiTi (GOAg) alloy.

**Figure 4:**
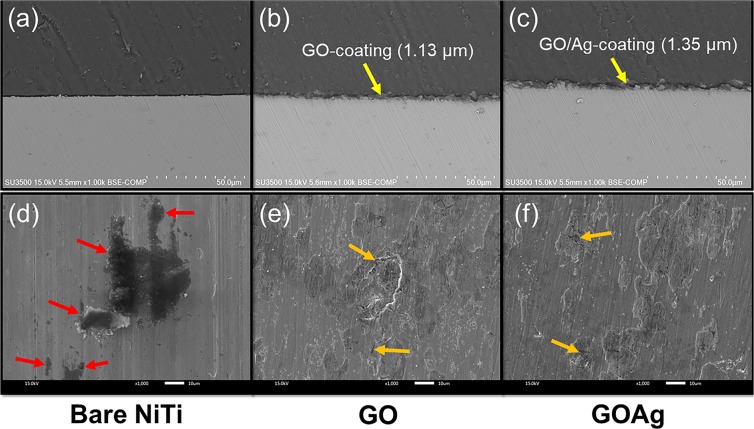
(**a**–**c**) The SEM analysis of the cross-section of the uncoated NiTi alloy, GO-coated NiTi alloy, and GO/Ag-coated NiTi alloy. A layer of GO-coatings (~1.13 µm) and GO/Ag-coatings (~1.35 µm) were evident on the surface of GO-coated NiTi alloy, and GO/Ag-coated NiTi alloy, respectively. Thickness of the coatings were analyzed from the surface profilometer. (**d**–**f**) The surface morphology of the bare NiTi GO-coated NiTi, and GO/Ag-coated NiTi alloys following the potentiodynamic polarization experiments. The arrows indicate the pitting corrosion.

The scanning electron microscope (SEM) images, energy-dispersive X-ray spectroscopy (EDS) analysis, and atomic force microscope (AFM) images of the bare NiTi alloy, GO-coated NiTi, and GO/Ag-coated NiTi alloy are shown in Fig. 2. The SEM images of the GO-coated alloy and GO/Ag-coated substrates show smooth and homogeneous morphology. Ni ions induce DNA damage and cytotoxicity in the body and Ti ions have mutagenic actions on human primary amnion cells^4^. The results from EDS analysis showed that the reduced Ni and Ti ions release from GO-coated and GO/Ag-coated NiTi compared to the bare NiTi alloy (Table 1). This implies that GO-coatings and GO/Ag-coatings increase the biocompatibility and decrease the release of these metal ions from the NiTi alloy. AgNPs interact with each other and form a triangular lattice by donating 1–2 electrons to the graphene^26^. From the Raman spectra analysis, the GO-coated NiTi displayed the D bands at 1344/cm and G bands at 1603/cm. Similarly, the GO/Ag-coated NiTi alloy displayed the D bands at 1340/cm and G bands at 1604/cm. The G band signifies the first-order scattering of sp^2^ hybridized domains of C atoms on the GO-coatings^27^. This confirms that the graphene coatings on NiTi alloy but not another form of carbon. The I_D_/I_G_ ratios were 0.83 for both GO-coated and GO/Ag-coated NiTi were consistent with other studies^28^. The GO-coatings were also confirmed from the XRD measurements. The peak observed at 12° was due to the stacking of the GO layers and it was absent in GO/Ag-coatings because of the presence of AgNPs on GO-coatings (Fig. 3). The presence of AgNPs in GO/Ag-coatings was also confirmed by the XRD measurements, i.e. the peaks observed at 2θ = 39° is attributed to the crystalline plane of AgNPs. From the surface profilometer, the mean thickness of the GO-coatings obtained was 1.13 µm and that of the GO/Ag-coatings was 1.35 µm as shown in Fig. 4a,b,c. As the thickness of single-layer graphene and GO can vary from 0.5 to 2 nm^29^, the GO-coatings and GO/Ag-coatings on NiTi alloys are multilayers and GO/Ag-coatings consisted mixture (nanocomposite) of GO and Ag in our study. The surface roughness (R_a_) determined from the AFM is 7.55 ± 1.7 for the bare NiTi, 11.67 ± 2.03 for the GO-coated NiTi, and 18.43 ± 2.43 nm for the GO/Ag-coatings NiTi alloys. Chembat *et al*.^30^ found the R_a_ of the bare NiTi alloy around 1.7 ± 0.1 nm. Similarly, Abdul *et al*.^31^ studied on the graphene composite coatings on carbon steel and found the surface roughness (Ra) were 34 to 150 nm according to deposition temperature where they measured using AFM. The difference in R_a_ values could be different due to the difference in the composition. In addition, the scan length/area used for the measurements of R_a_ from AFM may also affect the R_a_ values and longer scan lengths appeared to have resulted in a higher R_a_ value.

**Table 1:** Elemental analysis from energy-dispersive X-ray spectroscopy of the bare NiTi, GO-coated NiTi (GO), and GO/Ag-coated NiTi (GOAg) substrate.

Specimen	C (wt. %)	O (wt. %)	Ti (wt. %)	Ni (wt. %)	Ag (wt. %)
Bare NiTi	3.46	2.53	42.57	51.44	—
GO	12.68	5.53	38.21	43.58	—
GOAg	11.22	4.32	35.70	41.19	7.57

C = Carbon, O = Oxygen, Ti = Titanium, Ni = Nickel. Ag = Silver, and wt. % = weight percentage.

### Corrosion resistance

The surface morphology of the bare NiTi and coated samples following the potentiodynamic polarization experiments is shown in Fig. 4d,e,f. The SEM image of the bare NiTi surface shows the pits on the surface with the pitting corrosion as a result of the attack of the electrolyte (especially chloride ions) present in the medium as shown in Fig. 4d. Pitting corrosion is a common corrosion behavior found in NiTi alloy and the pitting usually starts at the site of defect in the protective oxide layer. At a critical potential (in the higher anodic region), the protective layer on NiTi breaks and the chloride ions invade into the interior surface of NiTi and dissociation of Ni and Ti into a soluble complex of Ni and Ti chloride takes place^24^. The pitting was absent in the coated samples with only few tiny areas of surface deterioration following the attack of chloride ions showing corrosion protection (Fig. 4e,f).

**Figure 5:**
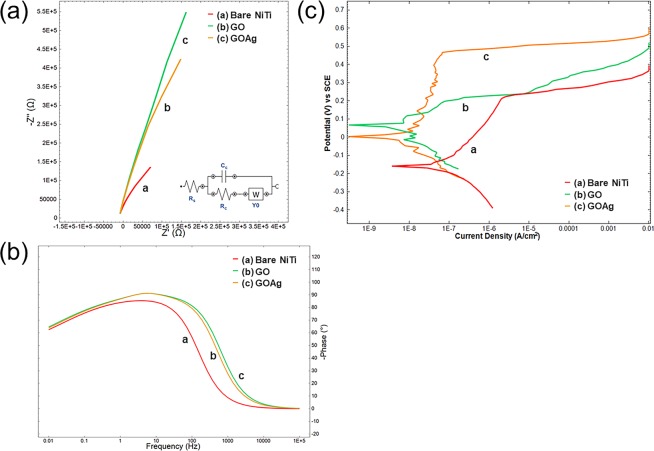
(**a**) Nyquist plots with equivalent circuit model, (**b**) Bode plots for the bare NiTi, GO-coated NiTi (GO) and GO/Ag-coated NiTi (GOAg) alloy, and (**c**) Potentiodynamic polarization curves of the bare NiTi, GO-coated NiTi (GO), and GO/Ag-coated NiTi (GOAg) after immersion in 3.5% NaCl. R_s_ = Electrolyte solution resistance, R_ct_ = Charge transfer resistance of the corrosion reaction, C_dl_ = Capacitance, W = Warburg Impedance related to the diffusion of O_2_ of the NiTi alloy, and SCE = saturated calomel electrode.

The Potentiodynamic polarization curves with equivalent circuit model and Bode plots of the bare NiTi alloy, GO-coated and GO/Ag-coated NiTi alloys are shown in Fig. 5. In our study, the Nyquist plots of the coated NiTi showed larger diameter than the bare NiTi alloy which represented higher corrosion resistance and lower corrosion rate. This is similar to the various other coatings, such as graphene/polyvinyl alcohol nanocomposite coating^24^, sol-gel film modified with GO^32^, and polyaniline/graphene composites^33^. The results of the electrochemical impedance spectroscopy (EIS) can be best fitted by the equivalent circuit model for both the bare NiTi alloy and coated substrates (Fig. 5a) and it consists of the electrolyte solution resistance (R_s_), the charge transfer resistance of the corrosion reaction (R_ct_), capacitance (C_dl_), and Warburg impedance (Y_o_), related to the diffusion of O_2_ of the NiTi alloy (W). Our equivalent circuit model was slightly different from another study^34^. Their model represented a two-layer of the oxide film including barrier-like inner layer and a porous outer layer. In our study, GO-coated NiTi and GO/Ag-coated NiTi did not show distinct two-layer oxide as the coatings were on NiTi alloy, hence, the model was different in our study. Also, the Bode plots showed the absolute value of impedance against the plotted phase shifts (Fig. 5b). The Bode plot showed less pronounced and frequency to measure the solution resistance. The electrochemical impedance spectroscopy (EIS) parameters of the bare NiTi alloy, GO-coated NiTi and GO/Ag-coated NiTi (Table 2). The mechanism of corrosion of NiTi is anodic oxidation which takes place at the working electrode along with the reduction process in cathode which utilizes the electrons released during the oxidation of Ni and Ti. Various anodic and cathodic reactions that take place on the respective electrodes are given below.

**Table 2:** Electrochemical impedance spectroscopy parameters of the bare NiTi alloy, GO-coated NiTi (GO), and GO/Ag-coated NiTi (GOAg) substrate.

Specimen	R_s_ (Ω)	R_ct_ (kΩ)	C_dl_ (µF)	Y_o_ (µMho)
Bare NiTi	16.20	16.80	36.90	8.29
GO	16.50	−6.12	26.60	15.60
GOAg	16.50	−5.04	47.20	23.20

R_s_ = Electrolyte solution resistance, R_ct_ = Charge transfer resistance of the corrosion reaction, C_dl_ = Capacitance, and W = Warburg impedance related to the diffusion of O_2_ of the NiTi alloy.

At the cathode: reduction of O_2_ as shown in Eqs. (1) and (2).

1 $${{\rm{O}}}_{2}({\rm{g}})+{{\rm{2H}}}_{2}{\rm{O}}({\rm{aq}})+{{\rm{2e}}}^{\mbox{--}}\to {\rm{2OH}}+{{\rm{2OH}}}^{\mbox{--}}({\rm{aq}})$$

2 $${\rm{OH}}({\rm{aq}})+{{\rm{e}}}^{\mbox{--}}\to {{\rm{OH}}}^{\mbox{--}}({\rm{aq}})$$

At the anode: formation of corrosion products as shown in Eqs. (3) to (6).

3 $${\rm{3Ni}}+{{\rm{6OH}}}^{\mbox{--}}\to {{\rm{3Ni}}({\rm{OH}})}_{2}+{{\rm{6e}}}^{\mbox{--}}$$

4 $${{\rm{Ni}}({\rm{OH}})}_{2}\to {\rm{NiO}}{{\rm{.H}}}_{2}{\rm{O}}$$

5 $${\rm{Ti}}+{{\rm{4OH}}}^{\mbox{--}}\to {{\rm{TiH}}}_{4}{{\rm{O}}}_{4}+{{\rm{4e}}}^{\mbox{--}}$$

6 $${{\rm{TiH}}}_{4}{{\rm{O}}}_{4}\to {{\rm{TiO}}}_{2}{{\rm{.2H}}}_{2}{\rm{O}}$$

The GO-coated NiTi and GO/Ag-coated NiTi restrict either the anodic oxidization or prevents the contact of OH^–^ ions. It is possible to inhibit the corrosion of NiTi. Besides, sufficient H_2_O and O_2_ are needed for the corrosion to take place and dissolution of the NiTi^33^. Hence, GO and GO/Ag-coatings play an important role in increasing the diffusion pathways of H_2_O and O_2_ molecules to the substrate.

Corrosion potential (E_corr_) and corrosion current density (i_corr_) are used to evaluate the corrosion resistance of a coating and they are obtained from the intercept of Tafel slopes. The polarization potential curves of the bare NiTi alloy and coated NiTi alloy substrates (Fig. 5c). It showed that GO-coatings and GO/Ag-coatings changed the processes of the cathodic O_2_ reduction reaction. This change has explained by the previous study^32^. Firstly, the good barrier property from GO layers and the increased cross-linking density of the coatings reduced the active surface area available for the attack of the corrosive medium which retarded the O_2_ reduction reaction. Secondly, the slight electrical conductivity of GO decreases the resistance of the active surface. The potentiodynamic polarization results of the bare NiTi alloy and GO-coated and GO/Ag-coated NiTi alloy substrates (Table 3). The E_corr_ measures the stability of the surface towards the corrosive environment. Compared to the bare NiTi, both GO-coated NiTi and GO/Ag-coated NiTi alloy substrates exhibited a positive shift of E_corr_, thus revealing the improved stability against corrosion of the bare NiTi alloy. The corrosion resistance behavior of GO-coatings and GO/Ag-coatings might be due to the barrier property and O_2_ functional groups on its basal planes and edges. Similarly, the i_corr_ of the GO-coatings and GO/Ag-coatings were lower than the bare NiTi alloy. Our results were corresponding to the other studies^25,35^. Cheng *et al*.^25^ found that graphene nanosheets improved the corrosion resistance of inorganic zinc-rich coatings due to the electrical conductivity of the graphene. In addition, the passive current density (i_p_) is shown in Table 3 which indicates the applied potential for the bare NiTi, GO-coated NiTi and GO/Ag-coated NiTi substrates. As compared to the bare NiTi, both GO-coated NiTi and GO/Ag-coated NiTi substrates showed lower i_p_ values. The lower values of i_p_ indicates the higher stability of passive film formed on its surface^36^. These supports the corrosion protective barrier of GO-coating and GO/Ag-coating as mentioned previously.

**Table 3:** Corrosion parameters of the bare NiTi alloy, GO-coated NiTi (GO), and GO/Ag-coated NiTi (GOAg) substrates.

Specimens	E_corr_ (V vs SCE)	i_corr_ (µA/cm^2^)	ʋ_corr_ (mm/year)	E_pit_ (mV)	i_p_ (µA/cm^2^)	η (%)
Bare NiTi	–0.170	0.158	16.06 × 10^−6^	0.214	0.626	—
GO	0.031	0.017	1.13 × 10^−6^	0.219	0.055	89.24
GOAg	0.008	0.002	0.02 × 10^−6^	0.467	0.040	98.73

Furthermore, the corrosion rate (ʋ_corr_) of the bare NiTi alloy was 1.606 × 10^−5^ mm/yr, whereas that of the GO-coated NiTi was 1.129 × 10^−6^ mm/yr and for GO/Ag-coated NiTi was 1.961 × 10^−7^ mm/yr. The corrosion rate of the bare NiTi in our study lies in between the results of other studies done by Zhang *et al*.^37^ (2.4 × 10^−4^ mm/yr) and Chembath *et al*.^30^ (6.98 × 10^−6^ mm/yr). Furthermore, Zhang *et al*.^37^ found that the corrosion rate of GO-coated NiTi alloy to be 1.4 × 10^−5^ mm/yr but in our studies the GO-coated NiTi showed better corrosion resistance (1.129 × 10^−6^ mm/yr) than theirs. The reason might be due to the use of low voltage (10 V) in their study than ours (30 V) for the coating using EPD. In addition, the GO/Ag-coated NiTi showed more corrosion resistance in our study which might due to presence of Ag which aided in the corrosion resistance. Additionally, the critical potential is the pitting potential (E_pit_), where the pitting nucleation starts. This change in corrosion behavior was visualized clearly for the bare NiTi alloy at 0.215 V (Table 3). But for the GO-coated NiTi and GO/Ag-coated NiTi, it showed more positive pitting potential. E_pit_ value was 0.365 V for GO-coated NiTi and 0.467 V for GO/Ag-coated NiTi. This indicates that both GO-coated and GO/Ag-coated NiTi presented higher resistance to pitting corrosion than bare NiTi alloy. Finally, the protection efficiency (η) in our study was up to 89.24% for GO-coatings and up to 98.73% for GO/Ag nanocomposite coatings.

Ag nanoparticles (AgNPs) have shown potent antimicrobial properties against several pathogenic bacteria and virus^9,38,39^ and they have wide biomedical applications, such as in medical diagnosis, wound sutures, surgical and prosthetic materials^40–42^. There have been some issues regarding the cytotoxicity of AgNPs on human cells but the toxicity of AgNPs is only seen at higher concentration^43^. In addition, AgNPs is only toxic in the suspension form at low silver ion fraction (5.5% fraction of 1.5 μg/ml Ag) than its supernatant^44^. Furthermore, *in vivo* oral exposure of AgNPs solutions at concentration of 10- and 32-ppm showed no clinical changes (in metabolic, hematologic, or urinalysis measures), and no morphological changes (in the lungs, heart or abdominal organs). Moreover, there were no significant changes in pulmonary reactive O_2_ species or pro-inflammatory cytokine generation^45^. Therefore, AgNPs with well-controlled application in human body is still an active area of interdisciplinary research. Considering this fact, we used AgNPs at a very low concentration (0.1 mg/ml) for the GO/Ag-coatings in our study and there would be no risk of Ag toxicity in human body if detached AgNPs from the coatings. Furthermore, because the main composition of the coatings is carbon and the Ag concentration used is very low, there is very low risk of galvanism from our GO/Ag-coatings due to dissimilar metals (presence of Ag with NiTi and other metals).

Although GBMs have wide coating applications^9,11,14–16^, the durability and stability are important concern for its long term use. Won *et al*.^46^ studied the durability and degradation mechanism of graphene coatings on Cu and found that a few layers thick graphene coating was effective in increasing the durability of the Cu substrate under dry contact sliding. The critical point of failure depends on the uniformity and the amount of defect of the graphene coatings. This implies that if a coating is uniform and less defect, it has high durability and stability. From our SEM images, the coatings were uniform and defect free, hence, we assumed our coatings to be durable and stable. But study on the durability and stability of the electrodeposited graphene coatings is needed before long-term clinical applications of the coatings.

Graphene based coatings can be produced from various methods such as chemical vapor deposition, electrophoretic deposition, spin coating, etc. Although there are certain disadvantages of graphene, such as, graphene is a catalyst is its susceptibility to oxidative environments and ecotoxicity of graphene, but still, GBMs have enormous potential for application in the field of protective coatings. Further improvements and development are going on for graphene-based coatings and various materials like polymers and ceramics have been combined with graphene to fabricate durable, stable and improved coatings^47,48^.

### Viability assay and immune response by human oral fibroblasts

**Figure 6:**
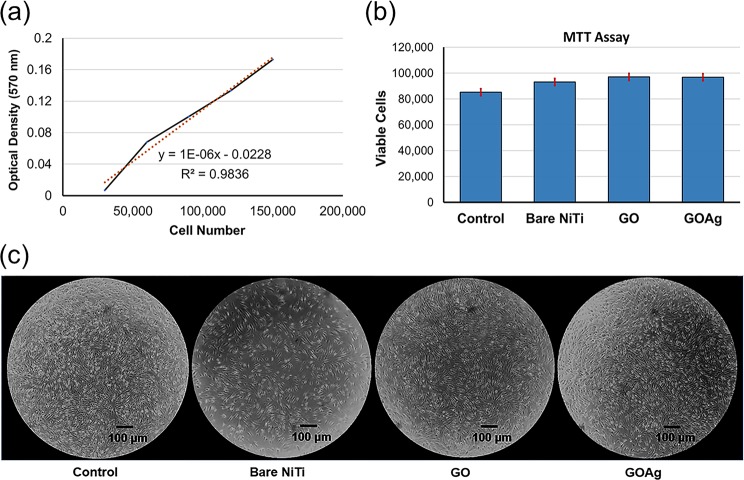
MTT assay of human pulpal fibroblasts. (**a**) Results of the standard curve of cell numbers, (**b**) cell viability, and (**c**) cell morphology visualized in the light microscope (at 20x magnification) of the bare NiTi, GO-coated NiTi (GO), and GO/Ag-coated NiTi (GOAg) alloy after incubating in the conditioned medium with 1% fetal bovine serum (FBS) for 24  hours. There was no significant difference in the viable cells among all the groups (P > 0.5).

Hence, studying the immune response of GBMs is an important part. The biocompatibility of the GO-coated and GO/Ag-coated NiTi on human pulp fibroblasts (HPFs) were studied previously by -[4,5-dimethylthiazol-2-yl]-2, 5-diphenyltetrazolium bromide (MTT) assay using culture media containing 10% fetal bovine serum (FBS)^22^. In this study, to test the viability of HPFs and expression level of IL-6 and IL-8, we used culture media with 1% FBS Dulbeccou’s Modified Eagle Medium supplemented with penicillin, streptomycin, amphotericin, L-glutamine (DMEM) to reduce the FBS induced cytokine production. Figure 6 shows the standard curve of the cell number, results of viability of the cells, and the cell morphology visualized in the light microscope after incubating for 24  hours in the conditioned medium of the bare NiTi, GO-coated NiTi, and GO/Ag-coated NiTi alloy. The cells were incubated for 24 hours assuming normally a material will release its maximum and active toxic products (if any) within 24 h and its toxicity decreases with time with decline in the concentration of the products^49,50^. In addition, the incubation period was kept 24 hours which was short enough to avoid reagent toxicity but long enough to provide adequate sensitivity^51^. The results showed that the number of viable HGFs showed no significant difference among the bare NiTi, GO-coated NiTi, GO/Ag-coated NiTi, and control (P > 0.5).

**Figure 7:**
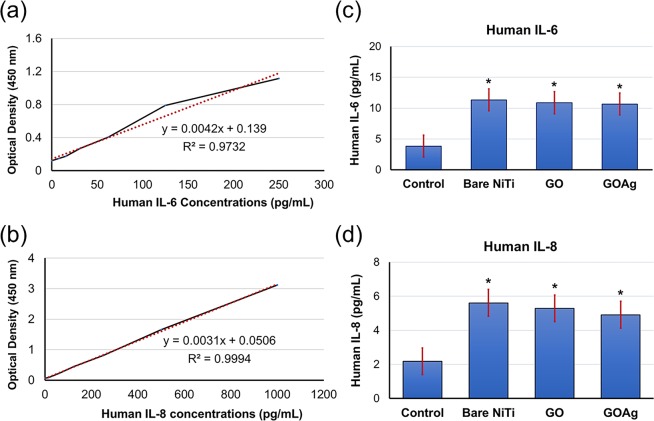
(**a**) Standard curve of ELISA human pulp fibroblast cytokines IL-6, (**b**) Standard curves of ELISA human pulp fibroblast cytokines IL-8, (**c**) ELISA measurement of cytokines IL-6 levels, (**d**) ELISA measurement of cytokines IL-8 levels. *Indicates a significant difference of cytokines IL-6 and IL-8 levels of the bare NiTi, GO-coated NiTi (GO), and GO/Ag-coated NiTi (GOAg) alloy compared to the control (P < 0.001).

The standard curves of IL-6 and IL-8 are presented in Fig. 7. The mean values of IL-6 were 3.848 ± 1.54, 11.349 ± 1.377, 10.872 ± 3.029 and 10.674 ± 1.036 (Fig. 7c) and those of IL-8 were 2.539 ± 1.176, 5.612 ± 0.612, 5.29 ± 1.35 and 4.913 ± 1.09 (Fig. 7d) in control, bare NiTi alloys, GO-coated NiTi, and GO/Ag-coated NiTi groups, respectively. The IL-6 and IL-8 levels of bare NiTi, GO-coated NiTi, GO/Ag-coated NiTi were significantly higher than the control (P < 0.001). The results are also similar to the results of Lategan *et al*.^52^ and Markhoff *et al*.^53^. The former studied on the effect of GO nanoparticles (GONPs) on the immune system and that GONPs stimulated IL-6 and IL-10 synthesis by whole blood cell cultures. Hence, they concluded that GONPs modulate immune system biomarkers and that may pose a health risk to individuals exposed to this type of AgNPs. While, the latter investigated the biocompatibility and inflammatory potential of four titanium alloys (NiTi, forged Ti6Al4V, additive manufactured Ti6Al4V, and DLC-coated NiTi) using osteoblastic cell line MG-63 as well as human osteoblasts, fibroblasts, and macrophages, and tested various cytokines including IL-6 and IL-8 using ELISA. They found that no distinct cell-specific response could be observed for the materials and surface coating used. This means the results of biocompatibility and inflammatory potential were not significantly different between uncoated alloys and surface modified NiTi alloys. Furthermore, it is also found that graphene may activate certain immune cells and on the other hand, causes suppression of maturation of immune cells^54^. This immunomodulation can be useful in a developing new vaccine, novel drug delivery, and biosensors^8,54,55^.

## Methods

### Synthesis of graphene oxide (GO) solution and GO/Ag solution

Graphene oxide (GO) powder with diameter 50 µm (Nanjing Jing Ji Cang Nano Technology Co., Nanjing, China) produced by a modified Hummers’ method was used in this study^56^. The GO was produced by a graphite oxidation process using potassium permanganate and concentrated sulfuric acid. A 100 ml GO solution (0.01 mg/ml) was prepared in deionized (DI) water and ultrasonicated for 3 h. A 100 ml silver nitrate (AgNO_3_) solution (0.1 mg/ml) was prepared and ultrasonicated for 20 min. The GO and AgNO_3_ solutions were mixed and ultrasonicated for 30 min. Finally, an AgNPs solution was produced by chemical reduction using trisodium citrate (Na_3_C_6_H_5_O_7_) as a stabilizing agent^57^. The GO/AgNO_3_ solution was heated at 80 °C for 1 h and 10 ml of Na_3_C_6_H_5_O_7_ (0.01 mg/ml) was added dropwise. The solution was maintained at 80 °C for 1 h and ultrasonicated for 20 min at room temperature to obtain a homogeneous GO/Ag solution. The potential of the suspension of GO solution and GO/Ag solution was measured by using a zeta potentiometer. A negative potential was found, i.e. −38 mV for GO solution and −39 mV for GO/Ag solution.

### Preparation of NiTi alloy samples

Medical grade NiTi alloy substrates consisting of Ni (55.8 wt. %), Ti (44.1 wt. %), and other elements (0.1 wt. %) (Baoji Seabird Metal Material Co., Ltd, China) were cut into 40 × 20 × 1 mm plates. The NiTi alloy substrates were polished with silicon carbide paper up to number 2000 to obtain a uniform roughness. The NiTi alloy substrates were ultrasonically cleaned by sequential immersion in acetone, ethanol, and deionized water for 10 min each. The substrates were placed in Kroll’s reagent consisting of 2 ml 40% nitric acid, 4 ml 40% hydrofluoric acid, and 994 ml deionized water for 10 min to remove any remaining oxide layer. Finally, the substrates were rinsed with DI water and dried.

### Electrophoretic deposition (EPD) of GO and GO/Ag

Freshly prepared NiTi alloy substrates served as the anode and the platinum metal served as the cathode. Two solutions were made; graphene solution and GO/Ag solution. The two electrodes were immersed in each solution parallel to each other 15 mm apart. Three EPD coating duration groups (n = 9) were prepared and the EPD was performed for 10 min with slow magnetic stirrer at a constant voltage of 30 V. After the EPD, the GO-coated and GO/Ag-coated were removed from the solution and rinsed with DI water, and dried at room temperature for 24 hours and kept at 80 °C for 3 hours.

### Characterization of GO-coated and GO/Ag-coated NiTi alloys

The microstructures of the GO-coated NiTi and GO/Ag-coated NiTi were observed in SEM (Quanta 250, FEI Co., Helsinki, Finland) at an operating voltage of 30 kV. The SEM analysis of cross-sections was done to visualize and verify the coatings on NiTi alloy. Elemental analysis of the coatings was performed using EDS (Quanta 250, FEI Co., Helsinki, Finland). A surface profilometer (Taylor Scan 150, Taylor Hobson Ltd., Leicester, UK) was used to evaluate the thickness of the surface coatings from the step height profile. An AFM (NX10, Park System, Suwan, South Korea) in contact mode was used to investigate the surface morphology. The Raman spectra were obtained using Raman spectroscopy (LabRAM HR Evolution, Horiba Scientific Inc., New York, USA) at room temperature with a laser at 532 nm with a grating of 1200 gr/mm and 200-μm slit. The crystallographic structures were analyzed by XRD diffractometer (AXS Model D8 Discover, Bruker AXS GmbH, Karlsruhe, Germany) operated at a generator voltage of 40 kV, and 40 mA. The data were collected in a 10° < 2θ < 90° range at a scan rate of 0.02°/step.

### Corrosion resistance

The corrosion resistance of the GO-coated NiTi and GO/Ag-coated NiTi were measured from a potentiostat with a standard three-electrode system using potentiodynamic polarization, and EIS measurement. Saturated calomel electrode, platinum electrode, and exposed NiTi alloy specimen with an area of 1 cm^2^ were made as a reference electrode, a counter electrode, and a working electrode, respectively. All measurements were conducted in a 3.5% NaCl solution at 27 °C using the µAutolab electrochemical workstation (µAutolab Type III, Eco Chemie, Utrecht, Netherlands) controlled by controlled by Autolab NOVA software 1.11.2. The EIS measurement was carried out at OCP in the frequency range from 0.01 Hz to 0.1 mHz with an AC amplitude of 0.01 V. The waiting time for the stable OCP was set to be 600 seconds before EIS measurement. Nova 1.11 software was used to analyze the EIS results. The polarization curves were obtained from the Tafel plots from linear sweep voltammetry (LSV). The sweeping range of potential was −0.25 V below OCP to 0.75 V above OCP and the potential scan rate was 1 mV/s. Through the intersection of the cathodic and anodic Tafel curves employing the Tafel extrapolation method, the electrochemical parameters, corrosion potential (E_corr_), and corrosion current density (i_corr_) and corrosion rate (ʋ_corr_) were calculated from the Eq. (7). The ʋ_corr_, equivalent weight *(EW)* of the, and η of the bare NiTi, GO-coated NiTi and GO/Ag-coated NiTi were calculated using the following expressions as per ASTM G102 − 89 standards^58^:

7 $${\rm{Corrosion}}\,{\rm{rate}}\,({\upsilon }_{{\rm{corr}}})=K\frac{{{\rm{i}}}_{{\rm{corr}}}}{\rho }EW$$

8 $${\rm{Equivalent}}\,{\rm{weight}}\,(EW)=\frac{1}{\sum \frac{{\rm{nifi}}}{{\rm{Wi}}}}$$

9 $${\rm{Protection}}\,{\rm{efficiency}}\,(\eta )=[{{\rm{i}}}_{{\rm{corr}}}({\rm{bare}})-{{\rm{i}}}_{{\rm{corr}}}({\rm{coat}})]/{{\rm{i}}}_{{\rm{corr}}}({\rm{bare}})\times 100 \% $$

where i_corr_ is corrosion current density, *K* is a constant that defines the unit of the corrosion rate (3.27 × 10^−3^ mm g/*μ*A cm year), i_corr_ is corrosion current density, *EW* is equivalent weight, and *ρ* is the density of NiTi alloy (6.45 g/cm^3^)^30^, fi is the mass fraction of the *i*^th^ element in the alloy or coating, Wi is the atomic weight of the *i*^th^ element in the alloy or coatings, and ni is the valence of the *i*^th^ element of the alloy or coatings, and i_corr_ (bare) and i_corr_ (coat) are corrosion current density for bare NiTi alloy and coated NiTi substrates, respectively. The passive current density (i_p_) were measured midway between E_pit_ and E_corr_. All the measurements were performed three times. The surface of some samples after the electrochemical tests were also examined.

### Cell culture

Human pulp tissues were obtained from the extracted third molars as described in the previous study^59^. Pulp tissues were collected from sound 3^rd^ molar teeth of healthy patients who were undergoing 3^rd^ molar extraction, and pulp cells were cultured in DMEM culture media^22^. The cells were cultured at 37 °C in a humidified 5% CO_2_ atmosphere. The culture medium was changed every 2 days. The cells were examined with a light microscope (Olympus, U-CMAD3, Tokyo, Japan). When the cells reached 80–90% confluence, the cells were sub-cultured using 0.25% trypsin-EDTA. The cell passage number 9 was used for this study. All cell culture media were purchased from GibcoBRLTM (InvitrogenTM, Grand Island, NY, USA). A total of 9 specimens of bare NiTi alloys (n = 3), GO-coated NiTi (n = 3), GO/Ag-coated NiTi (n = 3) of size; 3 × 5 mm were sterilized by autoclave.

### Viability assay and cytokines assay using ELISA

MTT assay was used to access the viability of HPFs using a method explained in the previous study^22^ with some modifications. We used 1% FBS DMEM culture medium instead of 10% FBS DMEM. A total of 30,000 cells/well were cultured using a 24-well culture plate (NuncTM cell culture plate, Thermo Scientific, USA). Each specimen was immersed in 5 ml of culture media containing only 1% FBS DMEM, and the control group containing 5 ml 1% FBS DMEM and all were incubated at 37 °C for 24 hours. Finally, the precipitated formazan crystals were dissolved in dimethyl sulfoxide (DMSO) and the optical densities were measured at an absorbance of 570 nm. The supernatants of the NiTi alloy, GO-coated NiTi, GO/Ag-coated NiTi, and control (cell culture only) were collected and stored at −80 °C for ELISA. The human IL-6 and IL-8 ELISA kits (Cloud-Clone Corp., Houston, TX, USA) were used to measure the released cytokine levels in the cell culture supernatants from bare NiTi treated, GO-coated NiTi treated, GO/Ag-coated NiTi treated, and control group. All experiments were made triplicated to confirm data and reduce variation among samples in each group. The cytokines results were determined using a microplate reader at a wavelength of 450 nm. The number of IL-6 and IL-8 were calculated using the standard curves for each IL type and the levels of IL-6 and IL-8 were expressed as pg/ml.

### Statistical analysis

The data were analyzed using the SPSS 18 for statistical software (SPSS, Chicago, IL, USA). Multiple comparisons were done using the One-Way ANOVA with Scheffe’s test at a significance level of 0.05.

## Conclusion

The GO-coatings and GO/Ag-coatings on NiTi alloy substrates were successfully developed by EPD. The mean thickness of the GO-coatings was 1.13 µm and of the GO/Ag-coatings was 1.35 µm. Both the GO-coated NiTi and GO/Ag-coated NiTi showed better corrosion resistance, a lower rate of corrosion, and higher protection efficiency than the bare NiTi alloy. Also, both the GO-coated NiTi and GO/Ag-coated NiTi were biocompatible to human pulp fibroblasts and showed upregulation of IL-6 and IL-8 levels.

### Approval

All experimental protocols were approved by the Ethics Committee of the Faculty of Dentistry, Chulalongkorn University (IBR number: DENT CU IBR 008/2019). Informed consent was obtained from the participants for this study.

### Accordance

The methods were carried out following the relevant guidelines and regulations.
